# The construction of a subset of ICNP® for patients with dementia: a Delphi consensus and a group interview study

**DOI:** 10.1186/s12912-015-0100-z

**Published:** 2015-10-06

**Authors:** Lene Baagøe Laukvik, Kathy Mølstad, Mariann Fossum

**Affiliations:** Department of Health and Nursing Science, Faculty of Health and Sport Sciences, University of Agder, PO Box 509, NO-4898 Grimstad, Norway; Norwegian Nurses Organization, Oslo, Norway; Deakin University and Deakin Alfred Health Nursing Research Centre, Alfred Health, Melbourne, Victoria Australia

## Abstract

**Background:**

The International Classification for Nursing Practice (ICNP®) 2013 includes over 4000 concepts for global nursing diagnoses, outcomes and interventions and is a large and complex set of standardised nursing concepts and expressions. Nurses may use subsets from the ICNP as concepts and expressions for research, education and clinical practice. The objective of this study was to identify and validate concepts for an ICNP subset to guide observations and documentation of nursing care for patients with dementia.

**Method:**

The process model for developing ICNP subsets was followed, according to the guidelines adopted by the International Council of Nursing (ICN). To identify relevant and useful concepts for the subset, a modified form of the Delphi method was used. Six nurses working in healthcare services in three municipalities in Norway with postgraduate education in geriatric psychiatry and dementia care participated in two Delphi sessions. The participants reviewed and scored the concepts included in the suggested subset and had an opportunity to rewrite them and offer alternatives. To validate the subset after the Delphi study, a group interview was conducted with six other nurses with postgraduate education in geriatric psychiatry and dementia care. The group interview was recorded and transcribed, and summative content analysis was used.

**Results:**

Suitable concepts for an ICNP subset to guide observations and documentation of nursing care for patients with dementia were identified. In total, 301 concepts were identified, including 77 nursing diagnoses, 78 outcomes and 146 nursing interventions. An increased focus on concepts to describe basic psychosocial needs such as identity, comfort, connection, inclusion and engagement was recommended by nurses in the validation process.

**Conclusions:**

Relevant and pre-formulated nursing diagnoses, goals and interventions were identified, which can be used to develop care plans and facilitate accuracy in the documentation of individuals with dementia. The participants believed that it may be difficult to find formulations for all steps of the nursing process. In particular, nursing diagnoses and psychosocial needs are often inadequately documented. The participants highlighted the need for the subset to contain essential information about psychosocial needs and communication.

## Background

Standardised nursing terminologies used in electronic healthcare records (EHRs) are important to improve content and quality of nursing documentation [1, 2]. Access to appropriate and reliable information is needed to ensure patient safety, continuity of care and communication with other healthcare professionals [3]. In clinical areas where patients, such as those with dementia, have limited ability to express themselves, an accurate EHR is essential for ensuring continuity and quality of care [3–5].

Terminologies in nursing care have evolved into large and complex sets of concepts. The International Classification for Nursing Practice (ICNP®) 2013 has approximately 4000 concepts to describe nursing. To use ICNP terminology in clinical practice, the development of five different types of catalogue is suggested: subsets, nursing minimum dataset, care plans, clinical templates and order sets [6, 7]. It is important that ICNP subsets are clinically validated and perceived as relevant and useful by nurses in given areas of nursing practice [7]. ICNP subsets may support nurses by providing concepts that are appropriate for documenting everyday nursing practices [8], which is the main focus of this study.

### Individuals with dementia and their basic needs

Nursing care for individuals with dementia entails compensating for their inability to fulfil their basic physical and psychosocial requirements. Prevention and management of pain, constipation, skin problems, malnutrition, physical exhaustion and unwanted side effects of medication are essential to physical care. Psychosocial aspects of basic care include safety, belonging and acceptance, the maintenance of social contacts and a sense of being able to contribute [9].

Person-centred care has been highlighted when developing frameworks for use in clinical practice to provide high-quality nursing care [10, 11]. Kitwood was the first to introduce person-centredness to dementia care [12, 13]. He described the five main needs of individuals with dementia as comfort, attachment, inclusion, occupation and identity. These needs overlap and meet in a central need for love [13]. Kitwood [13, 14] argued that individuals with dementia often exhibit an overt longing for love and have a generous, forgiving and unconditional acceptance towards people they meet. It is often argued that the concepts of personhood and person-centeredness are complex to translate into clinical practice [10]. McCormack [15] identified four core concepts of person-centred nursing; “being in relation, being in social world, being in place and being with self” and linked these to Kitwood’s definition of relationship. McCormack [15] argued that instead of striving for a particular status, it is important to focus on being more person-centred in clinical practice. We therefore suggest that it is necessary to take into account these elements concerning the needs of individuals with dementia when developing a subset of suitable nursing concepts.

### Nursing documentation

A focus on quality and safety in healthcare services has developed over recent years, and this has led to considerable demands relating to nursing documentation. Nursing documentation must ensure continuity of care, facilitate legal issues and support evaluations of the quality of patient care [16]. It reveals the decisions made by nurses in clinical practice, which are often based on nursing diagnoses, outcomes, interventions and an evaluation of the nursing process [1, 17, 18].

There is inadequate documentation of psychological, social, cultural and spiritual aspects of nursing care, while there is an excessive focus on biomedical parameters [17]. The change from paper-based nursing documentation to EHRs has increased the demand for a more standardised language when documenting nursing care [1]. Lack of structure in nursing documentation means that trends in nursing practices are difficult to identify, and because of the lack of access, nursing data are rarely used to support practice [19].

Despite widespread recognition of the importance of high-quality nursing documentation and efforts to improve it [1, 18], inconsistencies in its definition remain. This may be due to variations in practice based on differing requirements, documentation systems and terminology across locations and clinical situations [17]. Nurses rarely use pre-formulated concepts for nursing diagnoses, outcomes and interventions. Lack of standardised language and concepts may lead to difficulties in retrieving relevant patient information [1, 5, 17].

Nursing documentation includes terms and concepts that nurses use in daily practice. Without some form of classification or organisation of concepts, differences in nursing language may be quite pronounced, and this could lead to different interpretations of nursing documentation [19]. Standardised nursing terminology helps to eliminate ambiguities in language. This can promote improved communication within and across levels of healthcare, and may improve decision making [7]. Standardised nursing language can assist in providing common definitions and promote a common understanding and continuity of care. Standardisation also allows theory-based data to be collected and compared. This can provide opportunities for using recorded data in research [17]. Such data can also assist in the allocation of resources, the assessment of quality of care and policy-making in healthcare [20].

### ICNP

The ICNP is a classification system designed to be integrated into the global information infrastructure that informs healthcare practice and improves patient care worldwide. The set of concepts was created to facilitate expression of nursing diagnoses, interventions and patient outcomes [6, 7, 21]. It enables the construction of clinically-relevant, valid and useful standardised terminology in nursing practice, which is sensitive to cultural diversity and local conditions [19]. Concepts related to nursing diagnoses, goals and actions may vary for different subsets developed from the ICNP and are determined by nursing experts at individual sites. The categorisation of concepts is developed by nursing experts [6, 7, 21].

## Methods

This study follows the process model for the development of ICNP subsets, in accordance with guidelines adopted by the International Council of Nursing (ICN) [21]. A collection of concepts covering nursing diagnoses, outcomes and interventions was compiled. The concepts were formulated based on ICNP 2013, the evidence-based Practical Procedures in Nursing [22], and the clinical experience in dementia care of one of the authors.

A modified form of the Delphi method [23] was used to identify appropriate and relevant concepts. The method is highly valued for its ability to structure the consensus process [23, 24]. To validate the concepts in the subset, a group interview was conducted with nurses educated and trained in caring for individuals with dementia. It was thought that the group processes could help to explore and clarify participants’ views based on their extensive experience with individuals with dementia. The Norwegian Social Science Data Services (no. 34951) and the ethics committee of the Faculty of Health and Sport Sciences, University of Agder approved the study. Data were treated as confidential, and all personal information was removed.

### Sample and setting

A total of eight nursing homes in two municipalities in southern Norway were contacted by email. Five nursing homes providing long-term care participated. All the nursing homes had open units and lock-in units for individuals with dementia. No consensus has been reached about the recommended minimum numbers of participants in a Delphi study, but the quality of the participants is important [24]. Inclusion criteria were therefore nurses with specialised education in dementia care, more than two years of clinical experience working with individuals with dementia and experience using EHRs in nursing documentation. All nurses who were asked by the nurse unit managers agreed to participate in the study. The researcher contacted the nurses personally by email to ask them to sign a written consent form. Three coordinators of dementia care in three municipalities were invited to participate by email. These coordinators have the main responsibility for teaching healthcare staff and patients’ relatives about dementia in their respective municipalities. One coordinator of dementia care agreed to participate. In total, 12 nurses with specialised education in dementia care participated.

### Data collection

#### Sample Delphi study

The Delphi study group consisted of six nurses with postgraduate education in geriatric psychiatry and dementia care. The mean age was 45 years (SD = 5.9, range 39–53). Two participants had 0–5 years’ experience working with individuals with dementia, one had 6–10 years and three had 11–20 years. Two of the participants described their experience of nursing documentation using EHRs as expert, and four as intermediate. Three worked in locked units for people with severe dementia, and three in professional development and consultation on dementia care in nursing homes or in the municipalities.

#### Data collection

The researcher collected the signed consent forms and demographics personally. The Delphi study participants reviewed the set of concepts on two occasions. They were asked to judge, on a scale of 1–4, whether the concepts were relevant and useful for the documentation of nursing care for individuals with dementia. A score of one was “very relevant or very useful”, and four was “not relevant or not useful”. The first subset of concepts was sent by email as an Excel spreadsheet and received by the participants on 19th October 2013. The participants had two weeks to score them and suggest alternative formulations. The revised subset of concepts was emailed to participants on 29th November 2013, with a one-week deadline for scoring. All communication between the participants and the researcher was via individual e-mails. The first email to the participants contained in-depth information about how the concepts were organised in the subset and specified that this study did not seek to identify best practices, but to identify concepts that describe the needs of individuals with dementia when documenting nursing care.

In the first assessment, the participants judged a total of 368 concepts, including 93 nursing diagnoses, 114 outcomes and 161 nursing interventions. The concepts submitted for consideration during the second assessment were based on an analysis of scoring and review in the first Delphi session. The subset of concepts in the second assessment was reduced to 37 concepts which could not be agreement upon by consensus in the first assessment. During the data collection period, the participants were also able to contact the researcher by phone.

#### Data analysis

The aim of the analysis following the first and second Delphi sessions was to select concepts from the original directory or to identify new concepts formulated by the participants. The participant scores were calculated. To obtain agreement, the feedback was analysed using median and mean (Tables 1 and 2). This method was expected to yield accurate judgments because the questions sought nuances in each participant’s evaluation of the relevance and usefulness of each term. The purpose was to obtain the participants’ individual ratings, and calculate a preliminary agreement from the mean score for each term. Based on the mean score to one decimal place, the researcher determined the term’s relevance and practical usefulness in documentation of care for individuals with dementia. If agreement was reached in the first review, the term was not included in the second stage.

**Table 1:** Examples of concepts in the subset and participant scores in the first review of the Delphi study and calculation of median and mean agreement between the six participants

Term in the subset	Assessment of relevance^a^						Median score	Mean score
	Participant 1	Participant 2	Participant 3	Participant 4	Participant 5	Participant 6		
Urinary incontinence	1	1	1	1	1	1	1	1.0
Sleep deprivation	1	2	1	1	1	2	1	1.3
Impaired dentition	2	2	1	1	1	2	2	1.5
Ambivalence	1	3	2	1	1	2	2	1.7
Denial	2	2	1	4	1	2	2	2.0
Hyperventilation	2	4	2	2	1	3	2	2.3
Euphoria	4	3	2	1	1	3	3	2.3
Catatonia	1	4	4	4	1	3	4	2.8

^a^ Score 1 = very relevant, 4 = not relevant

**Table 2:** A selection of concepts in the subset and the process of agreement based on Delphi study reviews and scoring

Term in the subset	Median relevance	Mean relevance	Median usefulness	Mean usefulness	Level of agreement relevance	Level of agreement usefulness	Decision
Urinary Incontinence	1	1.0	1	1.0	High	High	Term kept No new assessment
Promoting recognition in the environment	1	1.3	2	1.5	High	High enough	Term kept No new assessment
Pain	2	1.5	1	1.3	High enough	High	Term kept No new assessment
Effective urination	2	1.8	2	2.0	High enough	High enough	Term kept No new assessment
Hyperventilation	2	2.3	2	2.2	Not high enough	Not high enough	New assessment
No social isolation	2	2.2	2	2.2	Not high enough	Not high enough	New assessment
Improved mobility in a wheelchair	3	2.5	2	2.3	Low	Not high enough	Term rejected No new assessment
Hypothermia	3	2.8	3	2.5	Low	Low	Term rejected No new assessment
Normal level of cholesterol	2	2.3	3	2.7	Not high enough	Low	Term rejected No new assessment

The level of agreement about the relevance and usefulness of each term varied, and the most common median agreement was 2 (the term is relevant and useful). Where there were variations in opinion, choices had to be made using the mean. To limit the directory to the key concepts, a decision was made that relevance would be given priority over usefulness in the selection of concepts. No opportunity to present alternative formulations was provided during the second review.

### Group interview

#### Sample group interview

The group consisted of six nurses with postgraduate education in geriatric psychiatry and dementia care. The mean age was 43 years (SD = 7.9, range 30–55). One nurse had 2–5 years of experience working in dementia care, two had 6–10 years, two had 11–20 years and one had over 21 years. Three participants described their experience with documentation using EHRs as intermediate level and three as expert. All were clinical nursing practice experts as suggested in the method for developing the terminology subsets of ICNP [7].

#### Data collection

Participants in the group interview were asked about their opinions of concepts that would be meaningful for nursing documentation for individuals with dementia. The participants received the sample of concepts via email a week before the group interview. It was important for participants to focus on every term in the different categories in the subset and consider whether the concepts were relevant and useful for the documentation of nursing care of individuals with dementia. The 90-min interview was conducted by two of the authors (LBL, MF) and recorded and transcribed verbatim. The structured interview guide contained open-ended key questions and the participants were asked to express their opinions about the concepts in the subset, and also suggest new concepts and amendments.

#### Data analysis

The transcribed text from the group interview was analysed using summative content analysis [25]. The analysis focused on the content and sought to describe the participants’ opinions of the sample of concepts. The text was condensed and summarised and summary areas were identified. Quotes are included to illustrate the participants’ opinions. Table 3 gives one example of the coding using summative content analysis.

**Table 3:** Example of summative content analysis from the group interview data

Transcribed text	Text unit	Condensed text unit	Category	Content category
I first thought that cognitive impairment is a main area and we focus a lot on it, making it very relevant	Cognitive impairment is a main area. It has the greatest focus and is most relevant	Cognitive impairment is a main area in dementia care	Cognitive impairment	Nursing Diagnosis
What is special with dementia is the cognitive impairment, all the other symptoms can apply to everyone else, so it is perhaps focus on the psychological and communication	Cognitive impairment is specifically for people with dementia, hence the focus on the psychological and communication	The greatest focus is on the psychological and communication	Psychological and communication	

## Results

### The Delphi study

The participants identified a subset of 301 concepts. They assigned a high value of relevance and usefulness to 77 nursing diagnoses (of which 53 related to physical needs and 24 to psychosocial needs), 78 outcomes (63 relating to physical needs and 15 to psychosocial needs) and 146 nursing inventions (115 relating to physical needs and 31 to psychosocial needs 31). These concepts were considered to be the most important concepts for nursing practice and documentation of individuals with dementia. Concepts related to physical needs clearly predominated in this subset. They also predominated among the concepts perceived as not relevant or useful, although for nursing outcomes, there were an approximately equal number of concepts related to physical and psychosocial needs. Examples of concepts are presented in Table 4.

**Table 4:** Examples of concepts from the subset; nursing diagnoses, nursing outcomes and nursing interventions

Nursing diagnoses	Nursing outcomes	Nursing interventions
Impaired self-feeding	Improved skin	Assisting with toileting
Urinary incontinence	Integrity	Assessing mobility
Diarrhoea	Positive appetite	Assessing medication side effect
Bowel incontinence	Improved status of teeth	Assisting with eating or drinking
Nausea	Effective self-hygiene	Assessing fluid intake
Chronic pain	Maintaining effective electrolyte balance	Assessing food intake
Impaired self-toileting	No pain	Facilitating adequate position when sitting/lying down
Impaired fluid intake	Improved impaired ability to perform hygiene	Assessing oral status
Self-care deficit	Improved ability to toilet	Assessing ability to swallow
	Self-effective urination	
Acute confusion	Improved perception	Assessing behaviour
Disuse	Dignified dying	Assessing discomfort
Chronic confusion	None euphoria	Calming technique
Impaired orientation	Reduced agitation	Reinforcing personal identity
Delusion	Able to communicate	Reminiscence therapy
Despair	None despair	Assessing cognition
Depressed mood	Reduced fear	Decreasing noises

### Group interview

The four summary categories identified in the data from the group interview were development of a care plan, nursing diagnoses, nursing outcomes for individuals with dementia and nursing interventions for individuals with dementia.

#### Development of a nursing care plan

The participants reported that specific concepts are particularly important in relation to cognitive impairment. The participants noted that the lack of knowledge about the healthcare of individuals with dementia and the lack of specific formulations may hinder documentation. One participant said, “In relation to the cognitive part, it is important to have statements, because there are assistants, and that is exactly where there is a lot of negative documentation about patients”. The participants also suggested that care should be documented in a manner that allows relatives and others to understand the content. One participant said, “I have often thought, as I wrote, that others may have access to this, and it is kind of important for me that I write it in such a way that it is okay for others to see”.

The participants noted that having a list of concepts to choose from may make it easier to remember all the information that should be included in the documentation. Specific concepts were preferred when developing a care plan. One participant said, “When I read through the list, I thought that it included so many concrete things. I feel you receive more information in the care plan, and that makes everyone think about it”.

According to the participants, documentation of nursing care for individuals with dementia should focus more on basic psychosocial needs and communication. Concepts related to comfort, attachment, inclusion, interests and identity should be specifically expressed in the care plan. Nurses can then apply greater focus to nursing care to meet psychosocial needs when developing a care plan. By documenting planned and performed nursing care, valuable information can be accessible to collaborating healthcare professionals, regardless of staff availability during a specific shift. The participants stated that receiving important information about individuals with dementia might happen simply by coincidence in clinical practice today. One participant said “we have so many examples of the things that have really worked time after time, but they are not written down”.

#### Nursing diagnoses for individuals with dementia

The participants mentioned that individuals with dementia experience behaviour changes that are similar to mental illness symptoms. They felt that it was important to distinguish between behavioural changes caused by dementia and those caused by mental illnesses. In particular, they mentioned relevant characteristics or symptoms in individuals with dementia as reduced language function, anxiety, despair, wandering, difficulty with spatial orientation, difficulty communicating different needs and a desire to be at home.

The participants noted that while physical, psychological, social, cultural and spiritual needs are all important for individuals with dementia, psychosocial and communication needs are most prominent. Physical needs are perceived as important for documentation because they may be difficult to communicate. The participants said that symptoms of anxiety often show that basic needs are not being met. When evaluating nursing care, focusing on the underlying causes was perceived as important. One participant described this as “What does she want to do now, why is she upset? Maybe she wants to go to the toilet, maybe she is hungry or thirsty?”

The participants perceived that many of the concepts in the physical needs subset were general concepts, appropriate for all patient groups. Participants noted the importance of basing observations and assessments on the need for protection of comfort, attachment, inclusion, interests and identity. They recommended additional and more specific concepts in the psychosocial needs subset. One participant expressed this as “…Kitwood’s flower. It is often forgotten, despite of the fact that this is where the focus should be. They are in the subset, but in minority compared to basic physical needs”.

There were different views on the concepts related to nursing diagnoses. Some participants thought that the concepts were too specific and needed to be simplified, keeping the list fairly short. Others reported that they found it helpful to have many specific concepts to choose from, especially in relation to nursing diagnoses and implementing relevant interventions. One participant said, for example, “There are a lot of variations around personal hygiene; they could be simplified a bit”. However, another said “I think when you have a list, it is easier to get everything important into the care plan”.

#### Nursing outcomes for individuals with dementia

The participants thought that when setting goals for nursing care, nurses should think about how an individual with dementia can best experience everyday life, and try to create good moments in everyday life. They thought nurses working with this patient group should try to make patients feel at home. One participant said, “There is something about the feeling of being at home amidst the feeling of chaos”.

The participants said that it is important for goals to be realistic and positively formulated. They believed that improving the situations of individuals with dementia and maintaining their daily life functions is realistic. One participant said, “there are not very many areas that we can improve a lot, a dementia diagnosis will take its course”, but another noted, “we have to maintain and preserve function for as long as possible”.

#### Nursing interventions for individuals with dementia

The participants suggested that follow-up of individuals with dementia may be inadequate if there is no specific documentation of nursing actions. The participants believed that many of the quotidian nursing interventions are only communicated orally, which means that only a minority of those involved in the patient’s care know about optimal follow-up. One participant said “they need to be performed similarly, the specific elements in the care plan, so that everyone can follow-up through all the shifts, not just the people that know it very well”.

The participants thought it was important to document small things in everyday life for individuals with dementia. They felt that interventions such as receiving a daily newspaper, routinely eating breakfast in the kitchen or in the bedroom and the location of personal equipment are important inclusions in care plans. The participants said that these small tasks are important in meeting the basic physical and psychosocial needs of individuals with dementia. They suggested that such interventions can highlight what is meaningful for each patient, how to comfort them and ensure their identity and/or best include them. One participant recommended that nurses should “be specific in their care plan and write that you can say ‘miss your mother’ if that particularly is reported by the patient, so one might instigate a conversation about the individual’s past”.

The participants believed that interventions that can provide a feeling of safety and security can ameliorate difficult situations, and a positive experience of recognition by the patient can induce feelings of calm. One participant said, “[…]when they turn around they see these faces and then they relax, they look on them as a mother figure. They feel at home because the nurses are caregivers they can trust”. According to the participants, confidence-building interventions can include looking at pictures from home, having a conversation about the past, especially about childhood, or reading a book that the patient appreciates. Another participant said, “Things that helped, I think, were photos, a calendar from where they had spent their childhood. That was so effective, plus she had book of poetry that calmed her very much when we read the poems to her”.

The descriptive interventions in the subset were judged by the participants as being important for providing safety and security for the individual with dementia. The participants felt that specific knowledge of how to manage particular situations involving individuals with dementia is essential. The participants note that planning appropriate interventions often required knowledge about the individual patient. One participant said, “if you look in the care plan at the interventions, you know what to do if a person is very restless and wandering around”.

Participants believed that therapeutic interventions have become so common that they are no longer considered to be treatment and are therefore not documented. They noted that accurate documentation could increase professional focus and involve more staff in the daily care of people with dementia. One participant said, “probably when you have worked with it [dementia] for years, the nice things that one does every day, that really are therapeutic things, are so common that you don’t see them as treatment”.

Some of the participants noted that limit-setting interventions are rarely used in dementia care, though others felt that they are sometimes necessary for special types of dementia diagnoses e.g. frontotemporal dementia, when limits can be reassuring. One participant said “in dementia care there are few limit-setting initiatives, except for special types of dementia”. Another said “because you have a large amount of insecurity, then maybe limits are a little safe too?”

The participants had different opinions of the concepts in the subset associated with nursing interventions. Some thought that the formulations in the nursing interventions subset were too long, while others found the long formulations more intuitive and precise. One participant said “I think that ‘Avoid explanations and questions that appeal to cognition’, is too long. But maybe it is explanatory”. Another said about the same formulation, “I think it is absolutely super. There is something about getting it on the spot”.

## Discussion

### The subset

Concepts related to physical needs dominated the concepts in the identified subset in diagnosis, interventions and outcomes. This is partly because the initial subset contained a clear predominance of concepts related to physical needs, but also because many individuals with dementia have difficulty communicating such needs, and therefore adequate concepts are required for the development of high-quality documentation and nursing care plans [9, 17].

Individuals with dementia often exhibit anxiety, and in many situations are unable to express their basic needs. Nurses should focus on communication and psychosocial needs when documenting nursing care for these patients [9, 17]. Concepts associated with psychosocial needs could be better represented in the identified subset, and participants recommended that more concepts related to comfort, attachment, inclusion, engagement and identity should be developed and integrated more thoroughly into the subset, in line with a person-centred approach [10, 13, 15]. Such interventions may be difficult to articulate for nursing experts [10, 15], but may add relevant concepts to the subset to support the daily documentation of nursing care for individuals with dementia. Expert nurses sometimes find it difficult to explicitly express their skilled practical knowledge as statements of principles or rules for action, as this embodied practical knowledge is complex. Formalising social, practical, local and historical bases in practice can be difficult [26]. Observation of nurses caring for individuals may give insight into these concepts, including more information about how they can be formulated.

The nursing diagnosis subset had the most concepts with a high consensus about their relevance and usefulness. This can be explained by the fact that nursing diagnoses are rarely formulated in long-term care [27]. As such, participants may not be used to describing the need for nursing care [5, 16]. The structure of a documentation system can also affect opportunities to formulate a precise nursing diagnosis [17]. The nursing outcomes subset had the largest proportion of concepts for which there was no consensus of relevance and usefulness. The participants felt that concepts such as “no” and “improved” are unrealistic when setting goals for individuals with dementia. A large proportion of the concepts in the nursing outcomes category had formulations such as “no pain” or “improved mobility”. These concepts should be reconsidered and reformulated to improve the subset. Nursing goals for individuals with dementia need to be realistic in relation to the disease and should seek to ensure that the individual feels satisfied in their daily life [9].

Approximately half of the concepts in the intervention category with high agreement on relevance and usefulness were related to psychosocial needs. This suggests that interventions to ensure safety and security are important for nursing care of individuals with dementia. To achieve care goals, it is important that the person feels safe. Nursing interventions should be reliable and seek to create good moments using positive recognition [9]. Specific and descriptive concepts for interventions that can provide safety and security for individuals with dementia can make it easier for everyone involved in their care to create a sense of inclusion and belonging, which will keep these individuals engaged and protect their identity [13].

The Delphi method proved suitable to identify relevant concepts for documentation of nursing care for individuals with dementia because of its flexibility, and the use of experts and group agreement as the basis for the findings. When developing ICNP catalogues, it is important that experts are involved and provide statements about suitable concepts for inclusion in directories [7, 21]. The participants in this Delphi study were given the opportunity in the first assessment to explain whether the concepts were relevant and useful and rewrite existing concepts or provide new ones. Participants assessed each term in the subset and were able to reconsider concepts without agreement. We therefore gained a good selection of concepts.

Further research should test and implement the subset for documentation in EHRs and explore whether it yields positive effects for individuals with dementia. Studies to investigate whether these concepts have positive effects on nursing documentation for individuals with dementia, and whether a subset helps nurses achieve appropriate care plans, are important. The identified subset should undergo the last two steps of the process model before they can be considered as a fully developed ICNP subset [21].

### Group interview

People with dementia are a particularly vulnerable group, often not able to communicate their needs [9, 28]. The study participants suggested a greater focus on concepts that describe basic psychosocial needs and communication such as identity, comfort, connection, inclusion and engagement. If such needs are assessed and documented, then individual perspectives can be documented in care plans and nurses may have more accurate information about patients with dementia [17].

Through the group discussion, different views and experiences of nursing care for individuals with dementia emerged and were used to validate the subset [24]. The findings from the Delphi study could be explored at a deeper level than in the written feedback from the participants in the two sessions of the Delphi study. The participants were highly-trained nurses with extensive clinical experience and knowledge of individuals with dementia. They could therefore provide important data to clarify whether the concepts were perceived as relevant and useful in their daily clinical practice. The group could also help to identify differences of opinion about the concepts.

## Limitations

The participants in the Delphi study did not have the opportunity to explain their opinions and clarify their statements orally, which might have affected the results. Another limitation may be that the initial list of concepts was not identified by the participants themselves [24].

The subset was quite extensive, and the time limit for feedback was short. This may have led to inaccurate replies from the participants. A longer time frame for data collection might have improved the quality of the replies.

Another limitation to the study is that the group interview did not include professionals from different disciplines, with different experiences of working with individuals with dementia. The number of participants was low, though the sample provided reliable data because the purpose of the study was to assess a limited subset of concepts from a nursing area. The results from the Delphi study were confirmed in the results from the group interview, which adds credibility to the study [29].

The participants were from five nursing services in two municipalities in southern Norway, and further studies should recruit a larger number of participants with a variety of backgrounds in nursing individuals with dementia. Several municipalities and a variety of nursing services could also be involved.

## Conclusions

This study identified concepts for an ICNP subset for monitoring individuals with dementia. The concepts in the subset were perceived as meaningful and relevant to nurses for the everyday practice of documenting nursing care for individuals with dementia. To safeguard the security, continuity and quality of nursing care for individuals with dementia, accurate information and a nursing care plan should contain concepts related to physical and psychosocial needs. Additional concepts regarding identity, comfort, attachment, inclusion and engagement should be identified and included in a further development of the subset.
